# Editorial: Community series in combinational immunotherapy of cancer: novel targets, mechanisms, and strategies, volume II

**DOI:** 10.3389/fimmu.2023.1256691

**Published:** 2023-08-11

**Authors:** Caili Xu, Dianwen Ju, Xuyao Zhang

**Affiliations:** Department of Biological Medicines & Shanghai Engineering Research Center of Immunotherapeutics, School of Pharmacy, Fudan University, Shanghai, China

**Keywords:** cancer immunotherapy, tumor microenvironment, biomarker, therapeutic target, chimeric antigen receptor T-cells

In recent years, cancer immunotherapies such as immune checkpoint inhibitors (ICIs), oncolytic viruses, and chimeric antigen receptor-T (CAR-T) cells have emerged as a promising approach for cancer treatment (1–4). However, the limited response rate and acquired resistance highlight the need for novel targets, mechanisms, and strategies to enhance therapeutic outcomes. Extensive research efforts have focused on identifying new immune checkpoints, exploring novel mechanisms of immune evasion, and developing innovative therapeutic strategies, such as combination immunotherapies and personalized immunotherapies (5–7). These advancements aim to overcome current challenges and improve the efficacy of cancer immunotherapy, paving the way for more effective and tailored treatment options for cancer patients.

In this topic, researchers investigated new biomarkers and therapeutic targets that could be applied to develop new diagnostic and therapeutic approaches. Ectonucleotide pyrophosphatase/phosphodiesterase 1 (ENPP1) is a membrane-localized glycoprotein that is highly expressed in various cancers and is associated with poor prognosis (8). Chu et al. isolated two potent human Fab antibodies against ENPP1 and developed effective therapies, including anti-ENPP1 IgG1 antibodies, antibody-drug conjugates, bispecific T-cell engagers, and CAR-T cells. These modalities showed promise as therapeutic candidates for ENPP1-expressing cancers. Liu Z. et al. investigated the role of coiled-coil domain containing 60 (CCDC60) in head and neck squamous cell carcinoma (HNSC). They found that CCDC60 expression was downregulated in HNSC tissues. High expression of CCDC60 closely correlated with favorable prognosis, diminished tumor growth and invasion, and immune cell infiltration, which provided a new promising biomarker and target for the diagnosis, therapeutics and prognosis of HNSC.

Research into the mechanisms of cancer onset, progression and treatment plays a key role in driving breakthroughs in cancer therapy, where many substances, pathways, and biological processes need a deeper understanding. Wang et al. summarized the regulatory effects of polyphenols on immune cells and their underlying mechanisms. Polyphenols can exert antitumor effects by enhancing the antitumor activity of NK cells, inducing the differentiation of myeloid-derived suppressor cells, inhibiting macrophage polarization to M2 type, and promoting the number and cytotoxic activity of CD8^+^ T lymphocytes. Several polyphenols can also induce immunogenic cell death and regulate Th1 cells and Treg cells. Recently, a large number of studies have indicated the significant role of m6A epigenetic modification in regulating cancer immunity (9, 10). Pan et al. conducted a comprehensive review on the regulatory mechanisms of m6A regulators in significant signaling pathways, including Wnt, P53, and PI3K/Akt, and their impact on immune checkpoint expression. These shed light on the functions and mechanisms of m6A epigenetic modifications in evading the immune response of tumors and offered valuable insights for future combination immunotherapy strategies. In addition, the researchers explored cancer biology and cancer treatment mechanisms from the perspective of ferroptosis. Ferroptosis represents an iron-dependent cell death that has the ability to directly kill tumor cells and therefore plays a crucial rule in tumor suppression (11, 12). In this Research Topic, the review by Qi and Peng primarily concentrated on the signaling networks and immune responses influenced by ferroptosis, especially emphasizing on the tumor microenvironment. The intersection of ferroptosis with iron metabolism, antioxidant metabolism and metabolism of the three major nutrients including glucose, lipid, and amino acid provides a vein for understanding the complex role of ferroptosis. The authors also discussed the innate and adaptive immune responses involving ferroptosis and presented innovative perspectives for advancing cancer research.

Based on the in-depth exploration of therapeutic targets and mechanisms, novel cancer immunotherapy strategies are emerging to increase efficacy and reduce toxicity. Severe toxicity is one of the most critical factors limiting the use of CAR-T cell therapies (13). In view of this, Saleh et al. developed a switchable Reverse CAR (RevCAR) T cell platform consisting of RevCAR T cells with extracellular peptide epitopes and a bispecific target module (RevTM) linking the peptide epitopes of RevCAR T cells and the tumor antigens. RevTM served as a switch key to activate RevCAR T cells and successfully achieved switchable and logic-gated activation of CAR-T cells targeting EGFR and GD2 in the treatment of glioblastoma. Additionally, Liu C. et al. investigated the side effect following the administration of ICIs through meta-analysis. They found that acute kidney injury (AKI) was observed in a considerable proportion of patients treated with ICIs, with an incidence rate of 5.7% and a median onset time of 108.07 days. There are many risk factors associated with AKI in individuals receiving ICIs, including advanced age, pre-existing chronic kidney disease, combination therapy of ICIs, and the utilization of proton pump inhibitors, which provides important insights into clinical treatment strategies.

In conclusion, the articles published in the Research Topic “*Community Series in Combinational Immunotherapy of Cancer: Novel Targets, Mechanisms, and Strategies: Volume II*” not only present potential targets for broadening our repertoire in modulating antitumor immunity but also explore the underlying mechanisms in tumor immunotherapy and propose strategies to enhance antitumor responses. These research holds promise for providing valuable boosts to the field of cancer immunotherapy.

## Author contributions

CX: Writing – original draft. DJ: Writing – review & editing. XZ: Writing – review & editing.
